# Neuroprotective Role of Klotho on Dementia

**DOI:** 10.7759/cureus.40043

**Published:** 2023-06-06

**Authors:** Fariha Noor Ananya, Md Ripon Ahammed, Simmy Lahori, Charmy Parikh, Jannel A Lawrence, FNU Sulachni, Tawfiq Barqawi, Chhaya Kamwal

**Affiliations:** 1 Internal Medicine, Dhaka Medical College and Hospital, Dhaka, BGD; 2 Research and Academic Affairs, Larkin Community Hospital, South Miami, USA; 3 Internal Medicine, Icahn School of Medicine at Mount Sinai, New York City Health + Hospitals/Queens, New York, USA; 4 Internal Medicine, Pramukhswami Medical College, Anand, IND; 5 Internal Medicine, Ross University School of Medicine, Bridgetown, BRB; 6 Internal Medicine, Liaquat University of Medical and Health Sciences, Jamshoro, PAK; 7 Internal Medicine, The University of Jordan, Amman, JOR

**Keywords:** aging, alzheimer’s disease, neuroprotection, dementia, klotho

## Abstract

Klotho, a gene found on chromosome 13q12, is involved in a variety of processes and signaling pathways in the human body related to vitamin D metabolism; cardiovascular, renal, musculoskeletal, and skin diseases; and cancer biology. However, more importantly, it has been linked to beneficial effects related to anti-aging. The levels of soluble Klotho in the blood have been found to decline with age, increasing the risk of age-related diseases. When the Klotho gene was silenced or defective, it caused a shorter lifespan. However, when the gene was overexpressed, it resulted in a longer lifespan. Klotho has positive benefits on the neurological system by causing a higher representation of useful longevity genes, preventing further neuronal damage, and offering neuroprotection. Thus, it has the potential to become a new treatment for many age-related diseases that cause dementia, including multiple sclerosis, Alzheimer’s disease, and Parkinson’s disease. In this review, we discuss the mechanisms of Klotho’s benefits and roles on various organ systems, specifically on nervous system disorders that lead to dementia.

## Introduction and background

Klotho is a membrane-bound anti-aging protein that functions as an endocrine, autocrine, and paracrine hormone targeting different cells [1]. There are two types of this protein in humans. One is a full-length membrane form associated with fibroblast growth factor receptors (FGFRs) and plays an active role in modulating phosphate and calcium homeostasis. The other is a soluble circulating form found in the blood, urine, and cerebrospinal fluid (CSF), where it regulates ion channels/transporters and growth factor signaling [2].

The Klotho gene is greatly exhibited in the kidneys, where it modulates renal function, parathyroid glands, and choroid plexus [3,4]. It plays a vital role in calcium-phosphate metabolism, remyelination, cognitive processes, and inflammatory processes [3,5]. In humans, the serum concentration of Klotho declines with age. Lower levels of serum Klotho can lead to stiffness in arteries resulting in vascular dysfunction, which, in turn, can assist in predicting atherosclerosis at earlier stages. Studies are demonstrating that a decline in the concentration of serum Klotho and expression of its gene in the thoracic aorta are associated with predicting the presence as well as the severity of coronary artery disease. The serum concentration of α-Klotho decreases with age, mainly after 40 years, leading to age-related diseases in humans, including cancer, hypertension, and kidney disease [6]. Klotho has also been found to exhibit anti-aging properties and plays a role in cognition. It causes changes in the structure of synapses in the hippocampus and cortex, resulting in slower cognitive decline [7].

Dementia is characterized by global cognitive impairment, consisting of an acquired decline in cognitive and emotional functioning, as well as behavioral and psychiatric disturbances severe enough to interfere with daily life. It is one of the most prevalent clinical syndromes seen in the old age group (age >65 years) [8]. It has a significant impact on performing daily life activities in the elderly, resulting in suffering, dependence, and a decrease in quality of life [9,10]. It can also be seen in the young aged <65 years, which is called pre-senile dementia [8]. Multiple studies have shown that the overexpression of Klotho can be beneficial to the aging brain and can have positive effects on neurodegenerative conditions [11-14]. The role of Klotho has been demonstrated in decreasing oxidative stress in neurons, promoting neuroprotection, and counteracting neurodegeneration [7]. Razani et al. (2007) summarized that it is Klotho’s involvement with calcium transport in the central nervous system (CNS) through the choroid plexus that explains the association between higher Klotho concentrations and better global cognition, as well as lower risk of cognitive decline [15].

In this article, we aim to further explore the associations between the Klotho gene and dementia and try to help build a platform so that work can be done to investigate the therapeutic potential of Klotho for neurodegenerative conditions such as Alzheimer’s disease.

## Review

Role, synthesis, and functions of Klotho in humans

Klotho, a gene present on chromosome 13q12, has three protein forms, namely, a secretory, a cell membrane-associated, and an intracellular form. Membrane protease can digest the extracellular Klotho domain and can be seen in CSF, urine, or blood [16]. Secretory Klotho acts as paracrine, autocrine, or endocrine hormones in target cells [17]. Klotho is found in organs such as the parathyroid gland, brain, skeletal muscles, pituitary glands, testes, kidneys, inner ear, ovaries, colon, and breast epithelial cells in its transmembrane form [18]. Klotho is associated with calcium, PO3, and vitamin D metabolism in the kidneys. Beta-Klotho (consisting of a β-glycosidase-like domain and sharing 42% amino acid sequence homology with Klotho) is seen in the kidneys, adipose tissues, liver, spleen, and gastrointestinal tract. Gamma-Klotho (a shorter type 1 single-pass transmembrane protein composed of a family 1 glycosidase-like extracellular domain and a short intracellular domain) is seen in the kidneys, eyes, and adipose tissues [1].

The circulating levels of soluble Klotho have been observed to decrease with age, which increases the risk of age-related illnesses. Torbus-Paluszczak et al. (2018) demonstrated accelerated aging and a shortened lifespan in mice when the Klotho gene was silenced or deficient [5]. In contrast, an extended lifespan was seen when the gene was overexpressed. Likewise, in humans, Klotho has been shown to display many beneficial effects, especially related to anti-aging. Although the membrane-bound Klotho protein was first associated with neurodegenerative diseases, it has been linked to various other age-related disease processes, including cancer biology and cardiovascular, renal, and skin diseases [19]. Klotho plays a role in cancer biology by serving as both a tumor suppressor and prognostic tumor biomarker, thus preventing and detecting neoplasms. Furthermore, Klotho overexpression (KL-OE) reduces the number of cancer cells that survive. Treatment with soluble Klotho has been shown to reduce tumor volume in preclinical cancer models in organs such as the stomach, pancreas, colon, and breast [19]. Concerning the link between cardiovascular illness and Klotho, Corsetti et al. discovered that decreased cardiac Klotho expression and increased cardiac fibroblast growth factor (FGF) expression lead to higher cardiovascular risk [20]. Klotho can be used as an early and sensitive biomarker for kidney illnesses, as well as a potential treatment for both acute kidney injury and chronic kidney disease (CKD) [21]. It is also protective against ultraviolet B (UVB)-induced damage, and its overexpression can considerably alleviate the UVB-induced damage to cells, an effect which can be seen with aging [9].

Expression of Klotho in the nervous system

In the CNS, a secreted version of Klotho plays an important role in neuronal activity. It is a critical component of processes such as aging and neurodegeneration. In CSF, the expression of cytokine/chemokine is suppressed by Klotho, thereby supporting the activation of neuronal cells.

Klotho is abundant in hippocampus neurons, choroid plexus ependymal cells, Purkinje EC cells, and the white matter of the brain [22]. Its relevance has been demonstrated for oligodendrocyte development and myelin integrity. It also guards against the formation of amyloid and glutamate neurotoxicity [23]. KL-OE is associated with considerably better cognition and memorization. The N-methyl-D-aspartate receptor (NMDAR) of the GluN-2B subunit is stimulated to generate this action. When Klotho levels are raised, it causes an increase in NMDAR-dependent genes, including Fos, which are involved in memory consolidation [24]. It has the potential to become a new treatment for demyelinating diseases, including multiple sclerosis and other degenerative diseases.

Role of Klotho on oxidative stress/free radical injury and age-related changes

Klotho was identified as a product of an anti-aging gene [25]. According to Smith et al., a genetic mutation in Klotho anti-aging protein is directly related to decreased life expectancy. It has anti-apoptotic, antioxidant, and neuroprotective activities [26]. These mutations affect molecular cascades directly related to chronic age-related diseases such as diabetic retinopathies, neurodegeneration, kidney diseases, tissue dysfunction, deficiencies in muscle regeneration, and mitochondrial functions. Multiple premature symptoms develop due to an insertion mutation in the α-Klotho gene, such as skin atrophy, motor neuron degeneration, organ atrophy, osteopenia, sarcopenia, atherosclerosis, thymic atrophy, vascular calcification, hypoglycemia, gonadal dysplasia, infertility, hyperphosphatemia, hearing impairment, and pulmonary emphysema, and overall life expectancy shortens, with these symptoms developing due to aging [1].

**Figure 1:**
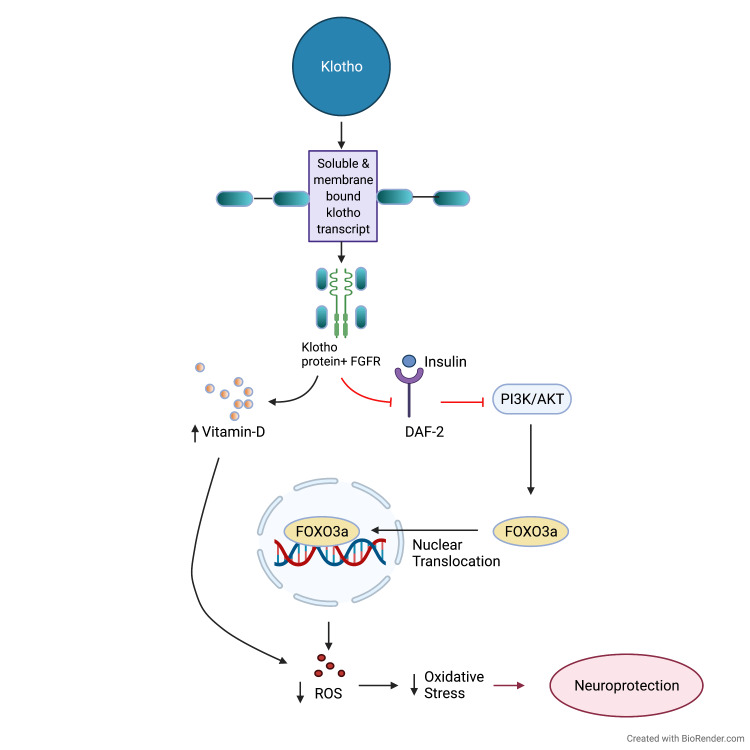
Mechanism of the Klotho gene on neuroprotection. FGFR: fibroblast growth factor receptor; DAF-2: dauer formation protein 2; PI3K/AKT: phosphatidylinositol 3-kinase/protein kinase B; FOXO3a: forkhead box O3a; ROS: reactive oxygen species

In contrast, KL-OE is expected to prolong the lifespan by reducing oxidative stress. It works like a hormone; thus, it attaches to the membrane receptor and prevents insulin-like growth factor-1 (IGF-1) from sending an intracellular signal [17]. Its concentration diminishes after age 40, resulting in cancer, renal disease, and hypertension [27]. According to researchers, for evolution and propagation of neural stem cells during switching to adult brain functionality, including postnatal neurogenesis Klotho. With age, Klotho expression and concentration are downregulated. Including cognitive deficits and oxidative stress, there is a direct connection between aging and neuropathological changes. Oxidative stress is one of the important mechanisms in the pathophysiology of dementia and Alzheimer’s disease. Klotho regulates oxidative stress, fibrosis, and inflammation and provides neuroprotection by inhibiting the signaling cascades of transforming growth factor-beta (TGF-β1) and IGF-1 [28]. Forkhead box O proteins are stimulated, and the expression of superoxide neutralizers such as manganese superoxide dismutase (MnSOD) is increased when the insulin/IGF-1/P13K (P13K/Akt) signaling cascade is suppressed. Klotho shows an anti-aging effect and resistance to oxidative stress [2]. By regulating the synthesis of vitamin D in the brain, Klotho affects the aging process. It inhibits the 1-α-hydroxylase enzyme, which catalyzes the 1, 25-dihydroxy vitamin D3 formation, the bioactive form of vitamin D. Therefore, vitamin D is a possible factor for the decline in age-related cognition. A schematic representation of the possible mechanism of neuroprotection is shown in Figure 1 [29].

Mechanism of action of Klotho and signaling pathways

Many biological activities of Klotho have been found; however, their molecular processes are unknown and must be explained. Research has shown that circulating Klotho has a role in intracellular signaling cascades by modulating the activity of multiple cell surface receptors, including TGF-β1 [18,19], p53/p21 [30], cyclic adenosine monophosphate (c-AMP) [31], protein kinase C (PKC) [32], Wnt signaling pathways [33], FGF-23 signaling [34], and insulin/IGF-1 [35]. Each signaling pathway that involves Klotho is described below in more detail.

Klotho’s Effect on the TGF-β1 Signaling Pathway

Several growth factors and cytokines induce epithelial-to-mesenchymal transition (EMT). TGF-1 is the most efficient and ubiquitous growth factor. It works by suppressing the expression of genes with epithelial activities such as E-cadherin and by increasing the production of genes with mesenchymal functions such as vimentin, collagen-1, and N-cadherin. TGF-1 signaling is disrupted by secreted Klotho, which binds directly to the type II TGF receptor and inhibits TGF-1 binding. Secreted Klotho inhibits EMT in cancer cells and prevents the spread of cancer [30,31].

Klotho’s Effect on the p53/p21 Signaling Pathway

Cell cycle arrest by tumor suppressor p53, which activates the downstream p53-dependent cyclin-dependent kinase factors p-16 and p-21, results in growth arrest and cell death. Klotho reduces cellular senility in MRC5 primary human fibroblast and human umbilical vascular endothelial cells by inhibiting the p53/p21-dependent pathway. According to one theory, Akt/protein kinase B (PKB) decreases cell lifetime via the p53/21-dependent pathway. However, these hypotheses need to be validated [30].

Klotho’s Effect on the c-AMP/PKA Signaling Pathway

c-AMP is generated from ATP by the enzyme adenylate cyclase (AC). c-AMP is required for protein kinase A (PKA). It stays catalytically inactive throughout the typical cell cycle. When c-AMP levels rise, an active form of PKA is released, phosphorylating and regulating several target proteins, including enzymes. By promoting superoxide generation, angiotensin-II (ANG-II) contributes to endothelial dysfunction. Membrane Klotho increases the angiotensin-converting enzyme (ACE) activity by stimulating the c-AMP/PKA pathway in human umbilical vein endothelial cells. Overexpressed Klotho suppresses the nicotinamide adenine dinucleotide phosphate oxidase 2 expression, thereby reducing ANG-II-induced oxidative damage, superoxide production, and apoptosis in smooth muscle cells. Hence, Klotho protects smooth muscle cells [31].

Klotho’s Effect on the PKC Signaling Pathway

PKC is an isoenzyme that forms a family related to serine/threonine kinases that regulate the target proteins. 25-OH vitamin D3 1-α-hydroxylase gene expression is regulated by both c-AMP and PKC. Klotho, on the other hand, inhibits the gene for 25-OH vitamin D3 1-α-hydroxylase in kidney cells. It is believed that Klotho suppresses the 1-alpha hydroxylase genes through a c-AMP or PKC-independent pathway. However, Klotho activates the c-AMP and PKC signaling pathways. PKC signaling pathway activation only occurs in the kidney and testes. In contrast, c-AMP activation occurs in each cell line [32].

Klotho’s Effect on the Wnt Signaling Pathway

Wnt1, a proto-oncogene, is an important secreted factor that is responsible for cell proliferation and its maintenance. Its continuous exposure leads to accelerated cellular senescence. The biological activity of Wnt is inhibited when it interacts with Klotho. This tumor suppressor effect of Klotho is due to the inhibition of Wnt signaling and p53/21 and insulin/IGF-1. Klotho plays a key role in tumor genesis, its progression, and its prognosis. In tumor cells, Klotho is silenced by histone deacetylation and by hypermethylation of its promoter. Low levels of Klotho are accompanied by raised levels of Wnt/beta-catenin, which lead to fibrogenesis. Consequently, the role of Klotho in CKD is evident, as it regulates Wnt/beta-catenin activity [33].

Regulation of FGF-23 Signaling Via Klotho

FGFR23 is a protein that targets the membrane expressions of Klotho and FGFR. FGF-23 initiates downstream signaling effects, such as GRB 2-associated binding protein 1 (GAB1), ERK1/2-SGK1, phospholipase C (PLC), Shc, fibroblast growth factor receptor substrate 2 (FRS2), or signal transducer and activator of transcription 1 (STAT1), through this complex, providing a likely mechanism for different genes to be expressed. According to some experiments, FGF-23 inhibits the biosynthesis of phosphate and vitamin D reabsorption in the proximal renal tubules. At the same time, its homozygous missense mutation in humans can lead to soft-tissue calcification due to severe hyperphosphatemia. Klotho and FGF-23, through apoptosis, prevent vitamin D-induced cell death and, through cell proliferation, prevent tissue atrophy. This signaling inhibits vitamin D-mediated cell death by downregulating 1-alpha hydroxylase gene expression and suppressing the caspase activity through phosphoinositide-3 kinase [34].

Klotho’s Effects on the Insulin/IGF-1 Signaling Pathways

The intracellular IGF-1 signaling cascade, which is blocked by the aging suppressor Klotho protein, extends lifespan. With Klotho binding, the insulin/IGF-1 receptor’s tyrosine kinase activity is blocked, followed by insulin receptor substrate inhibition (IRS). IRS inhibits PKB and phosphoinositol 3-kinase (PI3K) serine phosphorylation. The capacity of the Klotho protein to block the insulin/IGF1 receptor or the direct suppression of IRS, P13K, and PKB/Akt still needs to be determined. Klotho inhibits IGF-1-mediated signaling pathways, which suppress various tumor types. It stimulates the Foxo forkhead transcription factors, which are downregulated by insulin/IGF-1 signaling. Hence, it increases the mitochondrial MnSOD expression, eliminating the reactive oxygen species and providing oxidative stress tolerance in animals at the organismal and cellular levels. The Klotho hormone is also involved in oxidative stress resistance, which may add to its anti-aging properties [35].

Relation of Klotho deficiency and neurological abnormalities

With age, both the expression and circulating levels of klotho decline. In a study by Xiao et al., Klotho levels decreased with age in human blood serum samples [36]. Klotho deficiency has been associated with many neurological conditions, including multiple sclerosis, Alzheimer’s disease, amyotrophic lateral sclerosis, and Parkinson’s disease. The most common neurodegenerative condition is Alzheimer’s disease. Its neuropathology stems from the formation of neuritic plaque, which mainly consists of amyloid β (Aβ) protein further derived from amyloid precursor protein (APP). Semba et al. found significantly lower klotho levels in older patients than younger patients [37]. Another important discovery in that study was that patients with Alzheimer’s disease had lower CSF Klotho concentrations than those with normal cognition. Parkinson’s disease occurs due to the gradual loss of dopaminergic substantia nigra neurons and is characterized by worsening motor capabilities. Baluchnejadmojarad et al. demonstrated the neuroprotective effect of Klotho in a 6-hydroxydopamine rat model of Parkinson’s disease. Klotho prevented the degeneration of tyrosine hydroxylase-positive neurons in the substantia nigra pars compacta, which is key in the pathophysiology of Parkinson’s disease [38]. Both multiple sclerosis and amyotrophic lateral sclerosis are a result of an autoimmune demyelination event. Following cuprizone-induced demyelination, Zeldich et al. found that in transgenic mice overexpressing the transmembrane version of Klotho, the level of spontaneous remyelination was raised roughly two-fold [39].

Neuroprotective effect of Klotho in different types of dementia

Dementia is a neurological disorder categorized by impairment in memory in addition to one or more cognitive domains (e.g., language, personality, judgment). This impairment constitutes a decline from the previous level of function and should be significant enough to result in impairment in daily life [10].

It is estimated that about 47 million people suffer from dementia worldwide. As age is the most important risk factor leading to dementia, this number is set to increase in the coming years [40]. Other risk factors include sex, ethnicity, physical activity, smoking, alcohol use, level of education, and environmental and hereditary factors [41]. Alzheimer’s disease, vascular dementia, Lewy-body dementia, and frontotemporal dementia are the most common forms of dementia [42].

Alzheimer’s disease is characterized by extensive cortical atrophy caused by the deposition of hyperphosphorylated tau protein and amyloid plaques. Interleukin-6 (IL-6) is thought to activate microglia, which, in turn, leads to an increase in tau phosphorylation and the production of acute-phase reactions in neural tissue. A positive correlation was found between Alzheimer’s disease and elevated IL-6 levels [12]. Lewy-body dementia occurs due to the abnormal deposition of alpha-synuclein called Lewy bodies in the neurons. Accumulation of ubiquitinated TDP-43 and hyperphosphorylated tau proteins in the frontal and temporal lobes leads to frontotemporal dementia which is characterized by early cognitive abnormalities and speech problems. Vascular dementia is the consequence of cortical infarctions which lead to neuronal death [43].

Amyloid plaques in Alzheimer’s disease consist of β-amyloid which is created through the processing of APP. It is postulated that the extracellular processing of APP promotes Klotho expression which protects against the harmful effects of β-amyloid on the neurons. β-amyloid is also thought to damage neurons through reactive oxygen species, and Klotho has been illustrated to protect against these oxidative insults [4]. Higher levels of Klotho reduce Aβ plaques by 40-50% in the hippocampus and cortex of mice as well as reduce tau phosphorylation. Klotho has also been shown to inhibit the activation of the NLRP3/caspase-1 signaling pathway, a pathway stimulated by β-amyloid and thought to have an important role in Alzheimer’s disease. Additionally, KL-OE regulates the Aβ transporters which transport β-amyloid from the brain to the blood and prevent its accumulation [10]. A negative correlation has been reported between Alzheimer’s disease and the levels of Klotho protein in the CSF [37]. There is an association between lower Klotho levels and vascular dementia. Klotho protein is involved in the production of nitric oxide and the modulation of inflammatory processes which helps protect the endothelium [3].

Klotho contributes crucially to protecting hippocampus neurons from amyloid formation and plays a role against glutamate toxicity. Klotho is also considered key for oligodendrocytes maturation and myelin integration. The role of Klotho in the cellular life span regulation by p53 repression and downregulating p21 protein levels may be related to enhancing signaling through the insulin/IGF-1 pathway is already known [44]. On a microscopic level, persistent chronic inflammation, disordered cell proliferation, or cellular aging are responsible for several chronic diseases related to age, such as obesity, diabetes mellitus, atherosclerotic changes, Alzheimer’s disease (AD), malignancy, kidney diseases, and degenerative diseases.

Multiple Sclerosis

Multiple sclerosis is a demyelinating condition causing inflammation and myelin sheaths destruction in different parts of the CNS. A study showed that serum Klotho concentration is higher in multiple sclerosis patients with a longer disease duration when compared to the control group [45]. Zeldich et al. showed that the number of remyelinated axons was almost two times higher in mice with the KL-OE gene in comparison to the wild type [46]. Moreover, the remyelinated axons density was 1.76 times more in KL-OE mice. It illustrated a substantial beneficial role of the Klotho protein in remyelination. Thus, remyelination by Klotho modulation can be another potential target for the generation of new therapeutics for multiple sclerosis [47].

Alzheimer’s Disease

The disease process is centrally associated with the accumulation of amyloid plaques, neurofibrillary tangles, and neurodegeneration of various neuronal pathways of the brain. Multiple theories such as hyperphosphorylated tau proteins abnormalities and oxidative stress initiate the disease progression. A study done in Baltimore demonstrated that there are lower Klotho levels in CSF of Alzheimer’s disease compared to the control group, lower levels in older adults compared to younger adults, and higher levels in males than females [46]. Pretreatment of patients with Alzheimer’s disease with Klotho protein has been shown to decrease neuronal injury related to amyloid and glutamate. Hence, its use as a therapeutic strategy to provide neuroprotection in late-life depression, cognitive impairment, and dementia can be considered [23].

Cognition

All types of dementia are neurodegenerative, and protein toxicity is the principal feature. The leading causes are Alzheimer’s disease and Parkinson’s disease causing deterioration of brain functions. Klotho expression is reported in the adult hippocampus, and it enhances cognitive performance in mammals [44]. As mentioned earlier, Klotho acts as a neuroprotective protein by activating the antioxidant system and protecting the hippocampal neurons from the toxic effects of amyloid and glutamate. Moreover, it is necessary for oligodendrocyte maturation and myelin integrity [24]. A study conducted in California showed that the KL-VS variant improves cognition by increasing the secretion of Klotho. Higher Klotho levels correlate to better cognitive functions on the test [4].

Parkinson’s Disease

A study conducted in Iran showed findings denoting the neuroprotective role of exogenous Klotho in the rat model of Parkinson’s disease and the protective effect against astrogliosis, programmed cell death, and oxidative insults by different signaling cascades [48]. Zimmermann et al. demonstrated the association of CSF protein profiles with disease severity in Parkinson’s disease. CSF levels of Klotho and FGF-23 were lower in Parkinson’s disease patients than in the control group [13].

Role of Klotho in other organ systems

Reduced serum Klotho level has been linked to a greater density of epicardial fat, carotid artery intima-media, and reduced flow-mediated dilation of the brachial artery in the cardiovascular system; hence, reduced levels of serum Klotho must be addressed as a preliminary prognosticator of atherosclerosis [49]. The reduction of Klotho can uplift pro-oxidative, proinflammatory, and proapoptotic activity which leads to injury to cardiomyocytes making patients more susceptible to cardiovascular ailments. The capacity of cells to withstand the stress conditions can be elevated by increased levels of cardiac Klotho [20]. Klotho protein has been detected in the sinoatrial node pacemaker cells in mice and its representation is important for a stronger pacemaker activity of the sinoatrial node. Loss of function of the sinoatrial node and premature death was seen in mice with decreased Klotho levels [50]. Olejnik et al. showed that the balancing of serum Klotho and FGF level and their representation in cardiomyocytes could be indispensable for cell metabolism, correct functioning of the cardiovascular system, and defense in some different health conditions. It should be taken into account as a new essential element in cardiovascular ischemia such as myocardial infarction. The role of Klotho can be defensive in the injured myocardium and can be considered as therapy for cardiac disorders [51].

In the gastrointestinal system, the role of abnormal Klotho levels was observed in colorectal carcinoma. DNA hypermethylation specifically in the promoter region leads to suppression and notable downregulation of Klotho in malignant colon cells which leads to their multiplication. Adequate Klotho levels can cause apoptosis of malignant colon cells [52]. Klotho has a pivotal function in the suppression of tumors, and it also acts as a predictive tumor biomarker with an aim to help in the preliminary detection of cancers. Furthermore, Klotho overactivity causes a decline in the lifespan of malignant cells, and therapy with soluble Klotho leads to tumor density suppression in presymptomatic models of carcinoma [19].

In the renal system, Klotho deficiency is greatly linked with ionic imbalance, calcification in vessels, inflammation and fibrosis in the renal parenchyma, and skeletal disorders, which represents CKD. Stage 2 CKD shows a premature reduction in Klotho protein levels in the blood, and stage 1 CKD shows even an earlier premature reduction in urinary Klotho levels. Hence, deficient Klotho levels are responsible for the growth and succession of CKD and other complications. It shows an analytical capacity that acts as a demonstrative biomarker of renal disorders such as CKD [53]. Any treatment strategy that leads to an improvement in the Klotho levels by augmentation with external Klotho or uplifts the levels of endogenous Klotho manufacturing can be labeled a new novel therapy in renal disorders such as CKD [54].

Klotho is also found in the skeletal system, specifically in osteoblasts and osteoclasts. Klotho protein plays an important role as a co-receptor for FGF-23. It has an effective activity in the development of the skeletal system. Further research is required to use this discovery to form therapy for bone disorders such as osteogenesis imperfecta, osteoporosis, osteodystrophy related to renal system disorders, and other bone-related ailments [55].

Therapeutic role of Klotho on dementia

A randomized control trial on middle-aged healthy males conducted in Slovenia (2019) showed that a lower dose therapy of fluvastatin and valsartan causes a higher representation of useful longevity genes sirtuin-silent mating type information regulation 2 homolog, protein kinase AMP-activated catalytic subunit alpha, and Klotho, so it can be expressed as a favorable novel therapeutic perspective for senile disorders [56]. Dubal et al., in their animal interventional study, showed that higher Klotho levels and their efficient functioning cause a refinement in the synaptic and cognitive actions, so it can be beneficial in Alzheimer’s disease and other related disorders [24]. Pathogenesis of Alzheimer’s disease which predominantly includes damage to neurons associated with amyloid-β and glutamate can be prevented by preliminary therapy of neurons with Klotho protein. Hence, a major neuroprotective role is played by Klotho protein patients with Alzheimer’s disease. Increased levels of Klotho in the preliminary grades of the disease can express a curative master plan to reduce further damage and can also improve the consequences for Alzheimer’s disease patients who are older in age [46]. Older adults aged 60-80 years with normal cognitive function and the ability to carry APOE4 have a lower chance of AD and Aβ load with genotype KL-VSHET+. The KL-VS genotype should be taken into account in fusion with the APOE genotype to improve Alzheimer’s disease forecasting models that are accepted in various clinical studies and individualized genetic consulting [57].

## Conclusions

Klotho is one of the essential proteins that function as neuroprotective, anti-apoptotic, and suppresses oxidative stress. Since the detection of this protein, multiple studies have expanded our understanding of the significant role Klotho plays in the aging process. The levels of Klotho level decrease with age, which, in turn, increases the risk of developing dementia. Maintaining optimum levels of serum Klotho can slow this process. Furthermore, Klotho has been found to reduce neural damage and neurodegeneration, thus protecting against cognitive decline and contributing to life extension in humans. However, further studies are needed to explore its therapeutic role in humans.
